# Low Serum Total Calcium: Influencing Factors of Heart Failure with Preserved Ejection Fraction (HFpEF)

**DOI:** 10.3390/biomedicines14102194

**Published:** 2026-09-28

**Authors:** Na Li, Yuying Zhou, Jie Fan, Bin Peng, Kang Jiang, Yunlong Zhu, Dan Tan, Haobo Huang, Mingxing Wu, Jianping Zeng

**Affiliations:** 1 Department of Cardiology, The Central Hospital of Xiangtan (The Affiliated Hospital of Hunan University), Xiangtan 411100, China; sosnana258@163.com (N.L.);; 2 Department of Cardiovascular Medicine, The Second Xiangya Hospital of Central South University, Changsha 410011, China

**Keywords:** low serum total calcium, HFpEF, heart failure, outcome, serum calcium

## Abstract

**Aim**: The impact of low serum total calcium and the prognosis of patients with preserved ejection fraction heart failure (HFpEF) are not fully understood. This study aims to investigate the impact of low serum total calcium on the prognosis of hospitalized patients with HFpEF. **Method and Results**: This study included 9322 HFpEF patients (average age 72.04 years, 51.86% male), of whom 3408 had low serum total calcium. The patient completed a 1000-day clinical follow-up through telephone interviews, clinical follow-up, or community follow-up after discharge. The endpoints were heart failure (HF) readmission, all-cause mortality, and cardiovascular (CV) events. Multivariable standard Cox proportional hazards models and Fine–Gray competing-risk regression models were performed to evaluate the associations between total serum calcium levels and clinical outcomes. Specifically, Cox models were used for all-cause mortality while Fine–Gray models were applied to endpoints with competing risks, including cardiovascular events and heart failure readmission. This showed that low serum total calcium was an independent factor for HF readmission, all-cause mortality, and CV events and HR at 0.69 (95% CI: 0.61–0.77), 0.85 (95% CI: 0.78–0.92), and 0.65 (95% CI: 0.61–0.70), respectively (all *p* < 0.001). **Conclusions**: Low serum total calcium is an indicator related to a lower incidence of heart failure readmission, cardiovascular events, and all-cause mortality in HFpEF patients.

## 1. Introduction

HFpEF has become one of the most formidable challenges in contemporary cardiovascular medicine, owing to its high pathological and physiological heterogeneity and the rapidly increasing global prevalence [1,2]. At the cellular level, dysregulation of cardiomyocyte calcium cycling (characterized by impaired sarcoplasmic reticulum calcium reuptake and diastolic calcium leakage) is recognized as a central mechanism driving diastolic dysfunction and myocardial stiffness in HFpEF [3,4]. Regulation of sarcoplasmic reticulum calcium pump (SERCA2a) activity by microproteins such as DWORF [3] or modulation of calcium signaling pathways has shown initial impact on improving myocardial compliance and mitochondrial energy metabolism in HFpEF models [3,5]. A previous report showed that an increase in serum calcium level is associated with an increased risk of HFpEF in patients with type 2 diabetes [6]. Nevertheless, whether low serum total calcium serves as an independent outcome determinator remains to be defined. This retrospective study aims to analyze and evaluate the association between low serum total calcium and the outcome of HFpEF.

## 2. Methods

### 2.1. Study Population

This retrospective analysis included 9322 patients with HFpEF to investigate the prognostic impact of low serum total calcium(Figure 1). There were no patients with high serum total calcium (>2.6 mmol/L) in this cohort. Patients were stratified into low serum total calcium (<2.2 mmol/L) and non-low serum total calcium (2.2–2.6 mmol/L) groups based on serum calcium levels at first HF admission (Table 1). The diagnosis of HFpEF was established in accordance with the 2021 ESC Guidelines for the diagnosis and treatment of acute and chronic heart failure [7] and the 2023 ACC Expert Consensus Decision Pathway on Management of Heart Failure With Preserved Ejection Fraction: A Report of the American College of Cardiology Solution Set Oversight Committee [8]. The study was conducted in adherence to the principles of the Declaration of Helsinki and received formal approval from the Ethics Committee of Xiangtan Central Hospital. This study is registered with ClinicalTrials.gov (Identifier: NCT06487468).

### 2.2. Clinical Data Collection and Serum Biomarker Measurement

Clinical data are obtained based on the patient’s medical records, and the first serological test results after HF admission were collected and analyzed. This study did not calculate the albumin-corrected calcium. Total serum calcium was measured using standard laboratory automated analyzers, and low serum total calcium was defined as total serum calcium < 2.2 mmol/L.

### 2.3. Clinical Follow-Up and Outcomes

To minimize loss to follow-up and ascertainment bias, multiple strategies were employed. For patients who could not be reached via direct telephone contact or community visits, we implemented a rigorous tracking protocol by cross-checking their electronic medical readmission records and, ultimately, consulting the local police station (public security bureau) to verify their household registration and survival (vital) status. Loss to follow-up was meticulously tracked and compared between groups. As this is a retrospective study utilizing electronic health records, there is no need for active follow-up. On the contrary, the results were determined through an electronic database. During the initial queue selection period, a total of 19,297 patients with incomplete or ineligible tracking data were excluded, leaving 9322 participants with complete outcome data for final analysis. The endpoints were HF readmission, all-cause mortality, and CV events. The primary endpoint of interest was the cardiovascular composite outcome, defined as the time-to-first occurrence of cardiovascular mortality or unplanned heart failure readmission. Individual components, including heart failure readmission, were evaluated separately as secondary outcomes. Of note, heart failure readmission represents a constituent component of the cardiovascular composite outcome rather than an independent confirmatory endpoint, and these outcomes are interpreted hierarchically.

### 2.4. Statistical Analysis

Continuous data are presented as mean (SD) or median (IQR), depending on normality, which was assessed for all variables. Group differences were evaluated using the independent *t*-test or Mann–Whitney U test for continuous variables, and the Chi-square or Fisher’s exact test for categorical data. We assessed the association between total serum calcium and HFpEF endpoint events using multivariable Fine–Gray competing-risk models, with non-cardiovascular death treated as a competing event. To avoid potential selection bias and overfitting associated with traditional univariate screening, clinically relevant and predefined covariates were directly entered into the multivariable models. We reported both crude and adjusted SHRs with 95% confidence intervals (CIs). We visualized cumulative incidence through cumulative incidence functions and compared groups with Gray’s test. Serum calcium was analyzed as a continuous variable (mmol/L). Statistical computations were performed using IBM SPSS Statistics 28.0 and EmpowerStats 2.0. The proportional hazards assumption for the competing-risk regression models was evaluated, and any minor deviations were accounted for in the model structure.

## 3. Results

A total of 9322 patients were included in the final analysis and stratified into two groups (low serum total calcium group and non-low serum total calcium group). The incidence of low serum total calcium is 36.6% (*n* = 3408). The baseline clinical and laboratory characteristics are summarized in Table 1. Compared with the non-low serum total calcium group (*n* = 5914), patients with low serum total calcium were significantly older (71.7 ± 10.4 vs. 72.6 ± 11.0 years, *p* < 0.001) and more likely to be male (49.2% vs. 56.5%%, *p* < 0.001). From a laboratory perspective, the low serum total calcium group exhibited markedly higher serum creatinine (Cr) (117.1 ± 83.9 vs. 95.1 ± 56.3 µmol/L, *p* < 0.001) and elevated Ln N-terminal B-type natriuretic peptide precursor (NT-pro-BNP) levels (6.9 ± 1.4 vs. 6.8 ± 1.4 pg/mL, *p* = 0.018). Regarding comorbidities, the low serum total calcium group showed a higher prevalence of pleural effusion (20.0% vs. 13.5%), chronic obstructive pulmonary disease (COPD) (16.3% vs. 12.6%), and coronary heart disease (CHD) (70.3% vs. 61.0%) (all *p* < 0.001). Furthermore, electrolyte imbalances were more frequent in patients with low serum total calcium, including hypokalemia (14.2% vs. 11.1%, *p* < 0.001). 

### 3.1. Unadjusted Associations Between Various Clinical Parameters and the Three Primary Endpoints

The unadjusted associations between various clinical parameters and the three primary endpoints (HF readmission, all-cause mortality, and CV events) are summarized in Table 2. Univariate Cox regression analysis revealed that serum calcium (Ca) was a potent predictor across all outcomes (HR: 3.46 for HF readmission; HR: 2.21 for all-cause mortality; HR: 4.20 for CV events; all *p* < 0.001). Several other factors were identified as significant risk factors (*p* < 0.05) including Hemoglobin (Hb), Ln NT-pro-BNP, Pulmonary hypertension (PH), CHD, Renal insufficiency, Hyoxemia (Arterial oxygen level below 90%), Respiratory failure, Hypoproteinemia, Pleural effusion, Myocardial infarction, Furosemide, Torasemide, Nitroglycerin, Ezetimibe, Torasemide, Nitroglycerin, Ezetimibe, Methylprednisolone, and Dexamethasone.

### 3.2. Nonlinear Correlation and COX Regression Analysis

The smoothing spline plots (Figure 2) revealed a non-linear, piecewise relationship between serum calcium levels and the risk of adverse clinical outcomes in HFpEF. During a median follow-up of 1000 days, the cumulative incidences of adverse outcomes were consistently lower in the low serum total calcium group compared with the non-low serum total calcium group. The crude event rates for HF readmission were 31.07% in the low serum total calcium group versus 48.77% in the non-low serum total calcium group. Similarly, the incidences of CV events were 35.36% and 54.13%, respectively, and the all-cause mortality rates were 25.97% and 38.11%, respectively. To further evaluate the independent prognostic value of low serum total calcium, multivariable Cox proportional hazard models were constructed (Table 3). In the non-adjusted model, low serum total calcium was significantly associated with a reduced hazard of HF readmission (HR: 0.70, 95% CI: 0.65–0.75), all-cause mortality (HR: 0.85, 95% CI: 0.78–0.91), and CV events (HR: 0.64, 95% CI: 0.60–0.69) (all *p* < 0.001). After adjusting for potential confounders, including Gender, Age, Admission systolic blood pressure, D-2 Dimer, Glycosylated hemoglobin, High-density lipoprotein-Cholesterol, Ln NT-pro-BNP, Respiratory failure, Hypoproteinemia, Pleural effusion, Coronary heart disease, Pulmonary arterial hypertension, Renal insufficiency, Hypoxemia, Paroxysmal supraventricular tachycardia, and Dexamethasone, Prednisone remained as an independent predictor for lower incidence of all outcomes (Table 4). Specifically, the adjusted HRs for HF readmission, all-cause mortality, and CV events were 0.69 (95% CI: 0.61–0.77), 0.85 (95% CI: 0.78–0.92), and 0.65 (95% CI: 0.61–0.70), respectively (all *p* < 0.001) (Table 5). These results suggest that low serum total calcium is a critical and independent prognostic indicator for patients with HFpEF.

## 4. Discussion

This study reveals a clinically significant phenomenon in that low serum total calcium may serve as an indicator among HFpEF patients. Serum calcium levels are significantly positively correlated with the incidence of endpoint events, including all-cause mortality and HF readmission. Specifically, lower serum Ca^2+^ concentrations are independently associated with a reduced risk of endpoint events. This discovery indicates that low serum total calcium could be viewed as a factor influencing HFpEF patients.

However, our finding that low serum total calcium was associated with a reduced risk of adverse clinical outcomes in HFpEF is counterintuitive and warrants cautious interpretation. Furthermore, the divergence between crude event rates and adjusted multivariable Cox estimates underscores the presence of profound baseline confounding, which was successfully reconciled after rigorous multivariable adjustment for major clinical covariates.

Several potential explanations and methodological factors should be considered when interpreting these findings. First, clinical management bias cannot be entirely ruled out, as patients with lower calcium levels may have received closer monitoring or optimized medical therapy. Second, selection bias may exist, given that patients with severe comorbidities (such as end-stage renal failure or terminal malignancies) were excluded. Mechanistically, while profound changes occur in Ca^2+^ circulation in the setting of HF, it usually leads to impaired contractility and fatal arrhythmias [9]. The core pathological features of HFpEF involve decreased compliance and impaired active relaxation often linked to dysregulated cytoplasmic calcium removal (e.g., via SERCA2a imbalance) [10]. We hypothesize that lower extracellular calcium levels might theoretically attenuate this intracellular calcium overload, thereby improving myocardial relaxation and reducing filling pressures. However, because our study lacks direct intracellular measurements, these physiological considerations are strictly hypothesis-generating. Given the observational nature of this study, lower total serum calcium should be interpreted solely as a prognostic marker associated with favorable outcomes, and causal inferences cannot be established.

HFpEF is a systemic inflammation and microvascular endothelial dysfunction syndrome [6]. High concentrations of serum calcium are often associated with a higher risk of vascular calcification and increased arterial stiffness, such as increased pulse wave velocity [11]. Low levels of calcium may reflect a lower burden of systemic arteriosclerosis. The reduction in peripheral burden might have significant value for the fragile heart pump function of HFpEF.

Compared with previous studies, which showed that low calcium increases the HFrEF mortality rate [12]. The findings of this study show a significant “mirror” difference. In HFrEF, impaired systolic function requires higher Ca^2+^ to maintain stroke volume. Low calcium can directly lead to pump failure. However, in HFpEF, there is a relatively sufficient reserve of contractility. The main contradiction shifts to restricted relaxation.

Heart failure readmission constitutes a sub-component of the broader cardiovascular composite outcome. Therefore, the consistent risk trends observed across these endpoints reflect hierarchical alignment within the composite outcome rather than independent validation across disparate pathophysiological pathways.

A substantial number of patients (*n* = 12,189) were excluded from the initial screening cohort due to missing or incomplete echocardiographic data. This attrition is an inherent challenge in real-world retrospective studies, stemming from routine clinical documentation gaps, electronic medical record transitions, and untraceable external records, which may introduce a degree of selection bias and limit the generalizability of our findings. Several limitations of our study should be acknowledged. Due to the retrospective observational design, serum calcium was evaluated using total serum calcium at baseline, which may be influenced by serum albumin levels. Despite rigorous multivariable adjustment for major confounders and detailed tracking of follow-up status, residual confounding and unmeasured biases cannot be entirely ruled out.

The findings of this study provide new biomarkers for risk stratification of HFpEF. Monitoring of serum calcium levels helps provide a valuable prognostic indicator for HFpEF patients. Further studies are needed to validate this finding in other clinical cohorts.

## 5. Limitation

This study has several limitations. 1. This finding was derived from a single-center cohort. An external cohort study is needed to validate the result. 2. This study did not analyze related diseases (such as uremia, etc.) that affect blood calcium. 3. Calcium supplementation is common in elderly patients. 4. The lack of calcium or albumin-corrected calcium measurements in this study is a significant limitation in that it did not exclude the impact of hypoalbuminemia. 5. As an observational retrospective study, residual confounding remains a key limitation. Crucial parameters regulating calcium homeostasis—including serum albumin, parathyroid hormone (PTH), vitamin D, and ionized calcium—were not systematically available. Therefore, the observed inverse association should not be interpreted as evidence of direct clinical causality, and prospective studies with comprehensive metabolic profiling are required to validate these findings. 6. Serum calcium was measured only once at hospital admission, capturing only a single cross-sectional time point. We lacked data on serial calcium measurements or outpatient trajectories, preventing us from assessing whether persistent versus transient low serum total calcium differentially impacts long-term prognosis. Furthermore, because ionized calcium and comprehensive albumin-corrected data were limited in this retrospective setting, our findings should be strictly interpreted as associations with total serum calcium rather than active ionized low serum total calcium. Future studies are warranted to include subgroup analyses of HFpEF patients receiving long-term calcium supplementation versus those not receiving it.

## 6. Conclusions

In conclusion, our retrospective findings indicate an observational association between total serum calcium levels and post-discharge outcomes in patients with HFpEF. For clinicians, these results suggest that baseline serum calcium monitoring may aid in comprehensive risk stratification. However, given the observational design and potential unmeasured confounders, these findings should not be interpreted as evidence to alter standard clinical practices regarding calcium homeostasis or electrolyte correction. Future prospective trials with detailed metabolic profiling are necessary to determine whether managing serum calcium directly influences clinical trajectories in HFpEF.

## Figures and Tables

**Figure 1:**
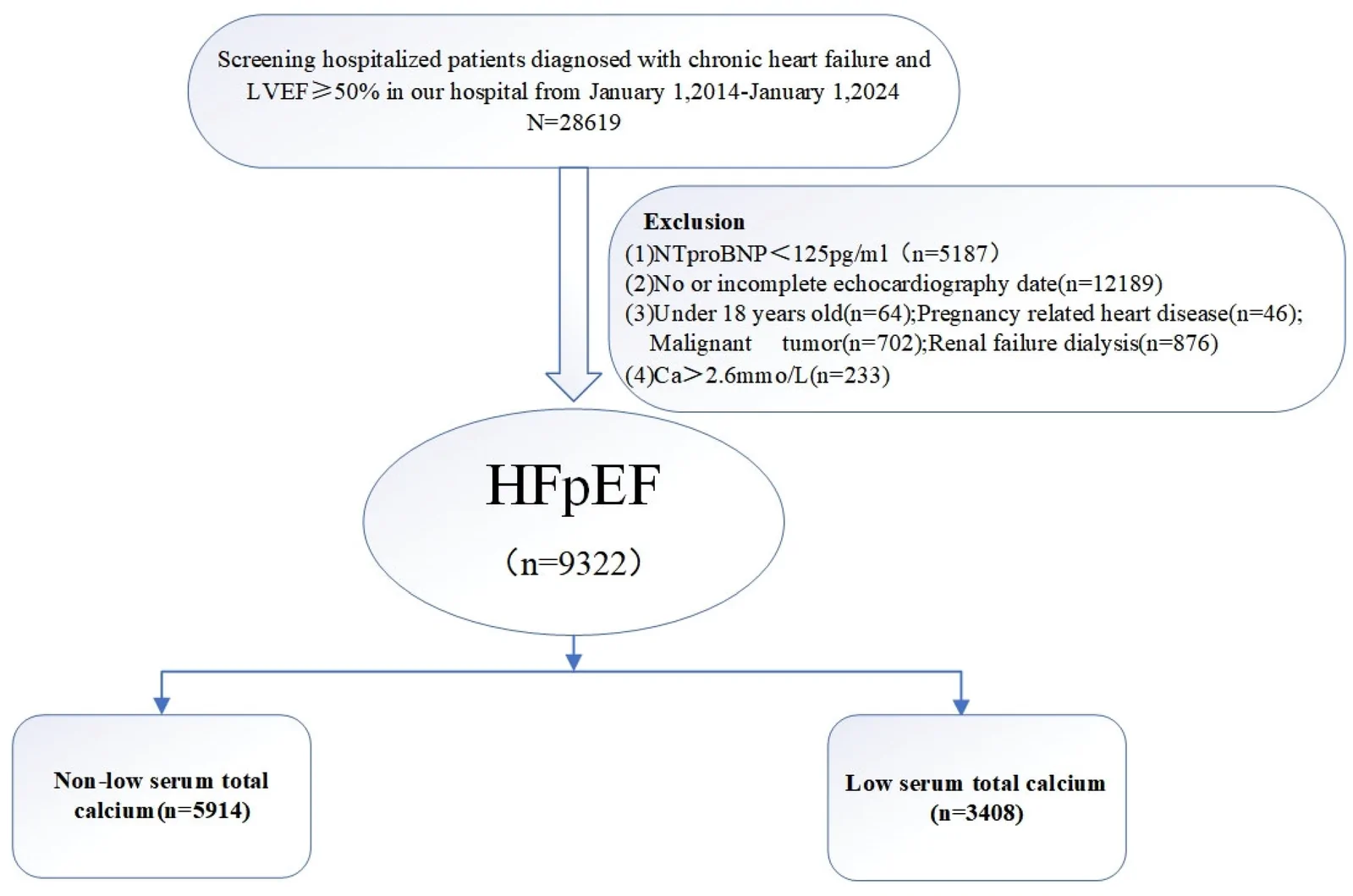
Flow chart of included patients with HFpEF.

**Figure 2:**
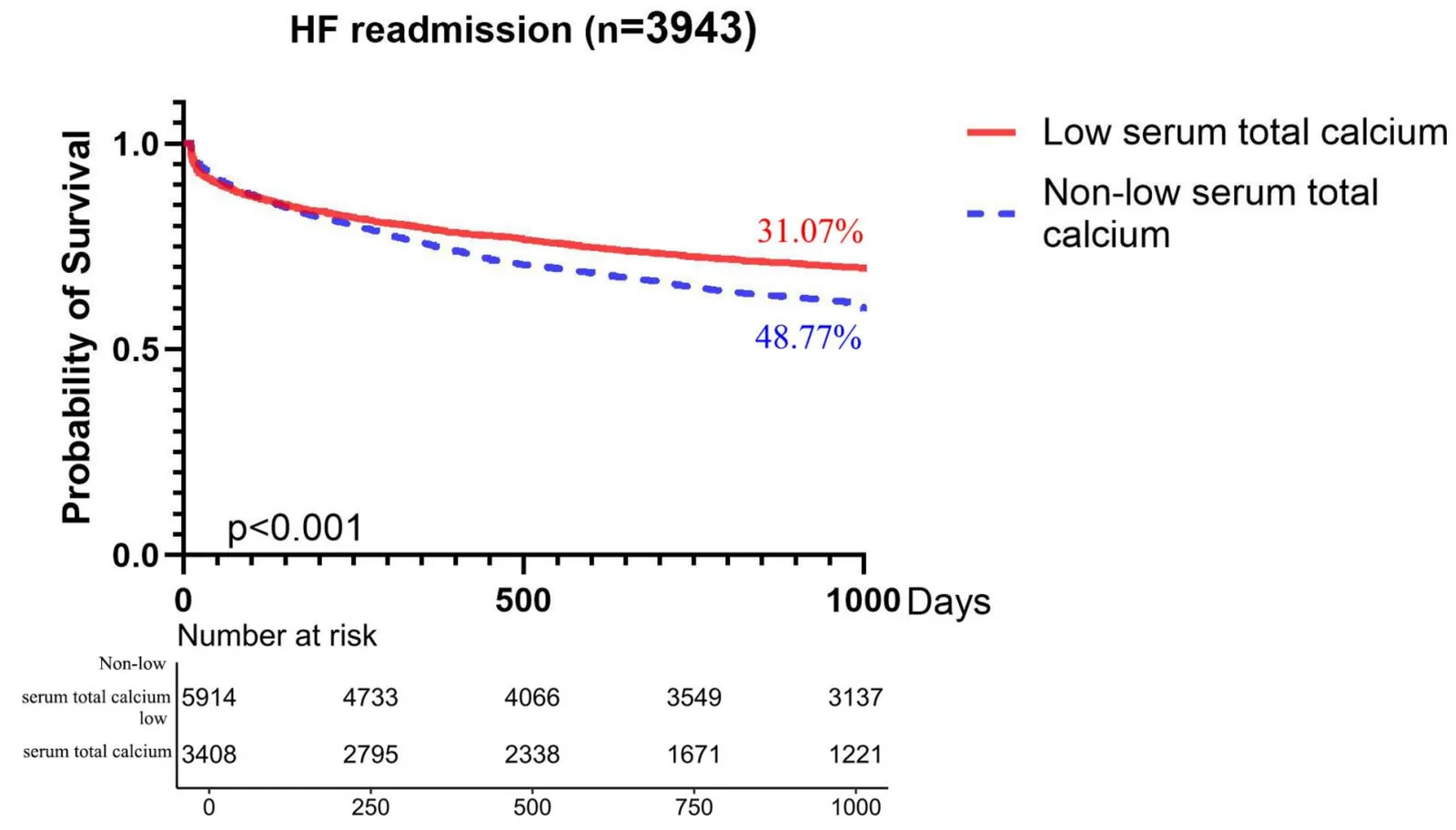

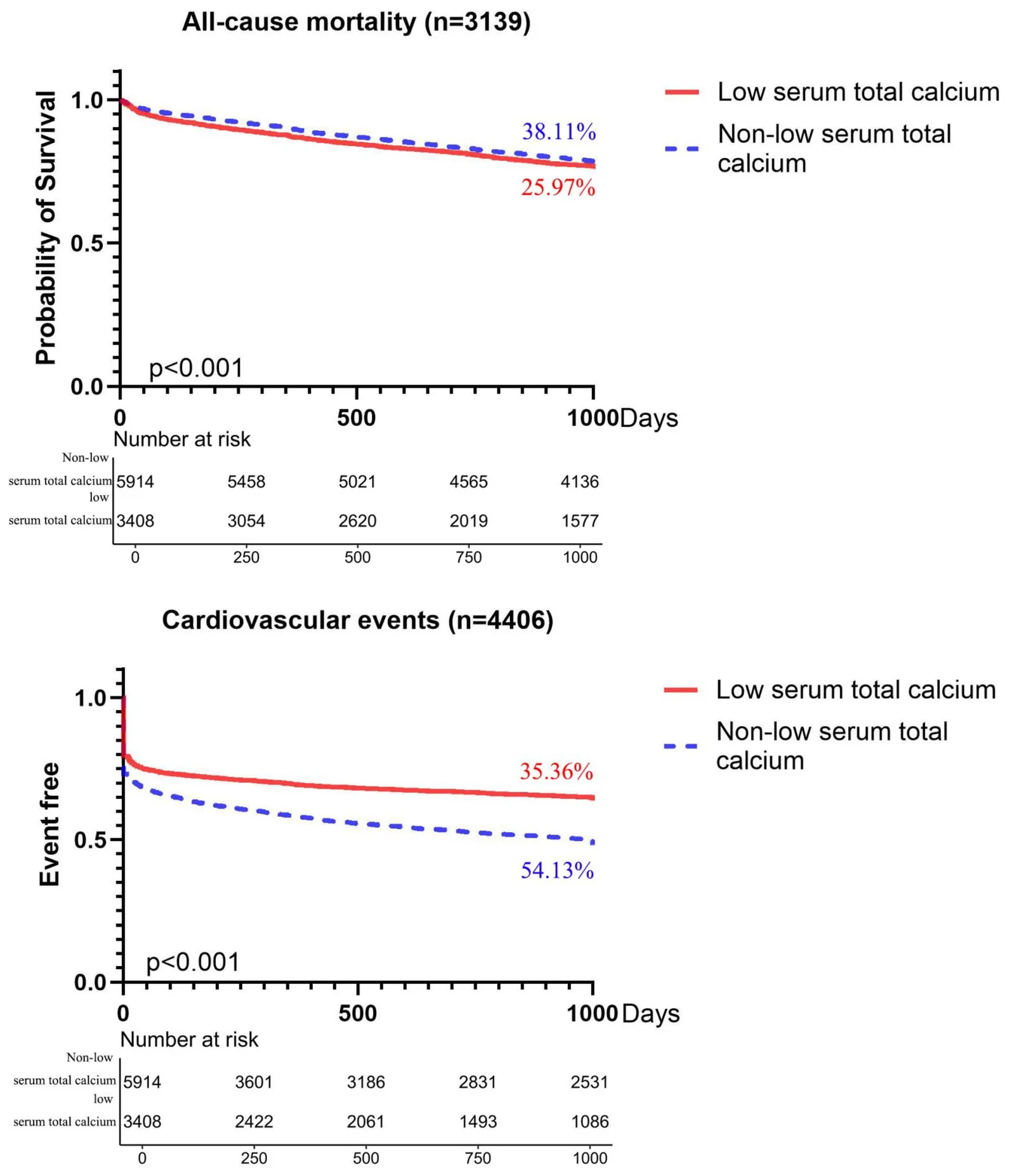
Kaplan–Meier analysis displayed the Cardiovascular events, Heart failure readmission, and All-cause mortality for patients with HFpEF between the low serum total calcium group and the non-low serum total calcium group. Abbreviation: HF: Heart failure.

**Table 1:** Baseline characteristics of the population stratified.

	Non-Low Serum Total Calcium	Low Serum Total Calcium	*p*-Value
N	5914	3408	
Age (years)	71.7 ± 10.4	72.6 ± 11.0	<0.001
BMI (kg/m^2^)	23.6 ± 3.6	23.3 ± 3.6	<0.001
Cr (μmol/L)	95.1 ± 56.3	117.1 ± 83.9	<0.001
Ca (mmol/L)	2.3 ± 0.1	2.1 ± 0.1	<0.001
LDL-C (mmol/L)	2.3 ± 1.0	2.4 ± 1.0	0.036
Ln NT-pro-BNP (pg/mL)	6.8 ± 1.4	6.9 ± 1.4	0.018
Gender			<0.001
Female	3004 (50.8%)	1484 (43.5%)	
Male	2910 (49.2%)	1924 (56.5%)	
Hypertension	4286 (72.5%)	2280 (66.9%)	<0.001
Pleural effusion	801 (13.5%)	680 (20.0%)	<0.001
Diabetes	1908 (32.3%)	1004 (29.5%)	0.005
COPD	748 (12.6%)	556 (16.3%)	<0.001
CHD	3608 (61.0%)	2395 (70.3%)	<0.001
Hypokalemia	657 (11.1%)	483 (14.2%)	<0.001
Hypochloridemia	411 (6.9%)	201 (5.9%)	0.048

Values are expressed as n (%)/Mean + SD. Abbreviation: BMI: Body mass index; Cr: Creatinine; Ca: Calcium; LDL-C: Low-density lipoprotein cholesterol; NT-pro-BNP: N-terminal pro-B-type natriuretic peptide; COPD: Chronic obstructive pulmonary disease; CHD: Coronary heart disease.

**Table 2:** Unadjusted risk factors associated with HF readmission, all-cause mortality, and CV events.

	Total	Heart Failure Readmission		All-Cause Mortality		Cardiovascular Events	
Gender		HR (95% CI)	*p*-Value	HR (95% CI)	*p*-Value	HR (95% CI)	*p*-Value
Female	4488 (48.14%)	1		1		1	
Male	4834 (51.86%)	0.95 (0.90, 1.02)	0.1361	1.00 (0.93, 1.07)	0.9943	0.96 (0.91, 1.02)	0.1999
Age (years)	72.04 ± 10.60	1.00 (1.00, 1.00)	0.6646	1.00 (1.00, 1.00)	0.6897	1.00 (1.00, 1.00)	0.5812
BMI (kg/m^2^)	23.45 ± 3.63	1.00 (0.99, 1.01)	0.9757	1.00 (0.99, 1.01)	0.5168	1.00 (0.99, 1.01)	0.8755
Admission SBP	139.26 ± 24.50	1.00 (1.00, 1.00)	0.8712	1.00 (1.00, 1.00)	0.3113	1.00 (1.00, 1.00)	0.7384
HB (g/L)	118.62 ± 22.42	1.00 (1.00, 1.00)	0.0406	1.00 (1.00, 1.00)	0.0184	1.00 (1.00, 1.00)	<0.0001
Ca (mmol/L)	2.24 ± 0.16	3.46 (2.79, 4.29)	<0.0001	2.21 (1.72, 2.84)	<0.0001	4.20 (3.45, 5.11)	<0.0001
Cr (μmol/L)	103.13 ± 68.52	1.00 (1.00, 1.00)	0.0320	1.00 (1.00, 1.00)	0.0386	1.00 (1.00, 1.00)	0.0003
D-2Dimer (μg/mL)	1.56 ± 2.81	0.99 (0.97, 1.00)	0.0411	1.00 (0.98, 1.01)	0.6438	0.98 (0.97, 0.99)	0.003
Glycosylated hemoglobin (%)	7.01 ± 1.71	1.01 (0.98, 1.03)	0.6534	1.00 (0.97, 1.03)	0.9015	1.00 (0.98, 1.03)	0.7291
HDL-C (mmol/L)	1.15 ± 0.38	1.01 (0.93, 1.09)	0.8703	1.15 (1.05, 1.26)	0.003	1.02 (0.95, 1.10)	0.5885
LnNT-pro-BNP (pg/mL)	6.82 ± 1.37	1.15 (1.12, 1.17)	<0.0001	1.42 (1.38, 1.45)	<0.0001	1.13 (1.11, 1.15)	<0.0001
PH	1490 (15.98%)	1.20 (1.11, 1.31)	<0.0001	1.44 (1.32, 1.57)	<0.0001	1.17 (1.09, 1.27)	<0.0001
CHD	6003 (64.40%)	0.84 (0.79, 0.90)	<0.0001	0.88 (0.82, 0.95)	0.0008	0.87 (0.82, 0.92)	<0.0001
Renal insufficiency	1331 (14.28%)	1.29 (1.18, 1.40)	<0.0001	1.92 (1.76, 2.09)	<0.0001	1.22 (1.13, 1.32)	<0.0001
Diabetes	103.13 ± 68.52	1.00 (1.00, 1.00)	0.0320	1.10 (1.02, 1.19)	0.0112	1.16 (1.09, 1.23)	<0.0001
Hypoxemia	870 (9.33%)	1.11 (1.00, 1.23)	0.0458	1.28 (1.15, 1.43)	<0.0001	1.13 (1.02, 1.24)	0.0166
Hypokalemia	1140 (12.23%)	0.91 (0.82, 1.00)	0.0621	1.25 (1.12, 1.38)	<0.0001	0.88 (0.80, 0.96)	0.0066
Hypochloridemia	612 (6.57%)	1.01 (0.88, 1.15)	0.9169	1.11 (0.97, 1.28)	0.1394	1.02 (0.90, 1.15)	0.7723
Respiratory failure	767 (8.23%)	1.28 (1.15, 1.43)	<0.0001	2.49 (2.25, 2.76)	<0.0001	1.24 (1.12, 1.38)	<0.0001
Hypoproteinemia	1866 (20.02%)	1.27 (1.18, 1.37)	<0.0001	2.36 (2.19, 2.54)	<0.0001	1.26 (1.18, 1.35)	<0.0001
Pleural effusion	1481 (15.89%)	1.27 (1.17, 1.39)	<0.0001	1.75 (1.61, 1.91)	<0.0001	1.20 (1.11, 1.30)	<0.0001
Supraventricular tachycardia	30 (0.32%)	0.71 (0.38, 1.32)	0.2791	0.49 (0.20, 1.18)	0.1136	0.78 (0.43, 1.41)	0.4104
Myocardial infarction	928 (9.95%)	0.77 (0.69, 0.87)	<0.0001	0.85 (0.75, 0.97)	0.0153	0.76 (0.69, 0.85)	<0.0001
Nitrendipine	10 (20.00%)	0.96 (0.36, 2.55)	0.9339	2.80 (1.23, 6.34)	0.0137	0.74 (0.28, 1.95)	0.5445
Furosemide	4421 (47.45%)	1.49 (1.40, 1.58)	<0.0001	1.83 (1.71, 1.97)	<0.0001	1.37 (1.29, 1.46)	<0.0001
Torasemide	1494 (16.03%)	1.56 (1.44, 1.68)	<0.0001	1.65 (1.52, 1.80)	<0.0001	1.35 (1.25, 1.46)	<0.0001
Nitroglycerin	1793 (19.24%)	1.32 (1.22, 1.42)	<0.0001	1.29 (1.19, 1.41)	<0.0001	1.24 (1.16, 1.34)	<0.0001
Ezetimibe	101 (1.08%)	0.49 (0.32, 0.76)	0.0013	0.50 (0.30, 0.84)	0.008	0.51 (0.35, 0.75)	0.0007
Methylprednisolone	1234 (13.24%)	1.23 (1.12, 1.34)	<0.0001	1.81 (1.66, 1.98)	<0.0001	1.17 (1.07, 1.27)	0.0003
Dexamethasone	353 (3.79%)	1.58 (1.36, 1.83)	<0.0001	1.41 (1.20, 1.66)	<0.0001	1.32 (1.14, 1.52)	0.0002
Prednisone	133 (1.43%)	1.30 (1.02, 1.66)	0.0331	1.32 (1.02, 1.72)	0.0377	1.24 (0.98, 1.55)	0.0692

Abbreviation: HR: hazard ratio; CI: Confidence interval; BMI: Body mass index; SBP: Systolic blood pressure; HB: Hemoglobin; Ca: Calcium; Cr: Creatinine; HDL-C: High-density lipoprotein-Cholesterol; NT-pro-BNP: N-terminal pro-B-type natriuretic peptide; PH: Pulmonary hypertension; CHD: Coronary heart disease.

**Table 3:** Multivariable COX model in HFpEF patients.

		Non-Adjusted		Adjusted ^a^	
	N	HR (95% CI)	*p*-Value	HR (95% CI)	*p*-Value
**HF readmission**		0.70 (0.65, 0.75)	<0.001	0.69 (0.61, 0.77)	<0.001
Non-low serum total calcium	2884				
Low serum total calcium	1059				
**All-cause mortality**		0.85 (0.78, 0.91)	<0.001	0.85 (0.78, 0.92)	<0.001
Non-low serum total calcium	2254				
Low serum total calcium	885				
**CV events**		0.64 (0.60, 0.69)	<0.001	0.65 (0.61, 0.70)	<0.001
Non-low serum total calcium	3201				
Low serum total calcium	1205				

Abbreviation: HR: hazard ratio; CI: confidence interval; HF: Heart failure; CV: Cardiovascular. ^a^ Cox proportional hazards regression models were adjusted for age; gender; admission systolic blood pressure; D-dimer; glycosylated hemoglobin; high-density lipoprotein-cholesterol; LnNT-pro-BNP; respiratory failure; hypoproteinemia; pleural effusion; coronary heart disease; pulmonary arterial hypertension; renal insufficiency; hypoxemia; paroxysmal supraventricular tachycardia; dexamethasone; and prednisone (all covariates were entered directly into the models).

**Table 4:** Subgroup Analysis and Effect Interaction Analysis in heart failure readmission.

	Non-Low Serum Total Calcium HR (95% CI)	Low Serum Total Calcium HR (95% CI)	*p*-Interaction
Female	Ref.	0.69 (0.62, 0.76)	0.6195
Male	0.96 (0.90, 1.04)	0.69 (0.62, 0.76)	
Age < 75 years	Ref.	0.69 (0.62, 0.76)	0.6114
Age ≥ 75 years	1.01 (0.93, 1.08)	0.72 (0.65, 0.79)	
Non-renal insufficiency	Ref.	0.69 (0.64, 0.74)	0.2678
Renal insufficiency	1.26 (1.13, 1.39)	0.96 (0.83, 1.11)	
Low Ln NT-pro-BNP (pg/mL)	Ref.	0.57 (0.50, 0.64)	<0.0001
High Ln NT-pro-BNP (pg/mL)	1.30 (1.21, 1.40)	1.04 (0.94, 1.14)	
Non-CHD	Ref.	0.76 (0.67, 0.85)	0.1852
CHD	0.89 (0.83, 0.96)	0.61 (0.56, 0.67)	

Abbreviation: HR: hazard ratio; CI: Confidence interval; NT-pro-BNP: N-terminal pro-B-type natriuretic peptide; CHD: Coronary heart disease.

**Table 5:** Multivariable Fine–Gray competing-risk regression analysis for heart failure readmission and cardiovascular events (with non-cardiovascular death as a competing risk).

	Heart Failure Readmission				Cardiovascular Events			
	SHR	95% CI Lower	95% CI Upper	*p* Value	SHR	95% CI Lower	95% CI Upper	*p* Value
Age (Years)	0.998	0.992	1.004	0.428	0.995	0.990	1.000	0.052
Gender	0.845	0.751	0.949	0.005	0.855	0.774	0.944	0.002
Admission systolic blood pressure (mmHg)	1.000	0.997	1.002	0.690	1.000	0.998	1.002	0.955
D-2Dimer (μg/mL)	0.967	0.936	1.000	0.049	0.981	0.957	1.006	0.126
Glycosylated hemoglobin (%)	0.987	0.952	1.022	0.459	0.996	0.966	1.026	0.777
High-density lipoprotein-Cholesterol (mmol/L)	1.137	0.941	1.373	0.184	1.013	0.868	1.182	0.875
Ln NT-proBNP (pg/mL)	1.474	1.221	1.779	<0.001	1.146	0.976	1.347	0.097
Respiratory failure	1.510	1.235	1.846	<0.001	1.223	1.028	1.455	0.023
Hypoproteinemia	1.741	1.366	2.219	<0.001	1.370	1.106	1.697	0.004
Pleural effusion	1.706	1.250	2.329	0.001	1.475	1.133	1.920	0.004
Coronary heart disease	1.173	0.962	1.432	0.116	1.123	0.947	1.331	0.182
Pulmonary arterial hypertension	1.387	1.201	1.602	<0.001	1.207	1.067	1.365	0.003
Renal insufficiency	1.197	1.024	1.399	0.024	1.062	0.926	1.219	0.390
Hypoxemia	0.927	0.818	1.051	0.235	0.917	0.826	1.018	0.104
Paroxysmal supraventricular tachycardia	0.994	0.847	1.167	0.944	1.002	0.871	1.154	0.973

Abbreviation: SHR: Subdistribution hazard ratios; CI: Confidence interval; N-terminal pro-B-type natriuretic peptide. SHR were calculated using multivariable Fine–Gray competing-risk regression models. Non-cardiovascular death was defined as the competing event for both heart failure readmission and cardiovascular events.

## Data Availability

The ethical restrictions and patient privacy regulations of Xiangtan Central Hospital prevent the authors from making the raw clinical data publicly available. However, the data that support the findings of this study are available from the corresponding author upon reasonable request, subject to institutional ethical approval.

## Abbreviations

HFpEF	Heart failure preserved ejection fraction
HF	Heart failure
CV	Cardiovascular
SERCA2a	Sarcoplasmic reticulum calcium pump
HR	Hazard ratio
CI	Confidence intervals
Cr	Creatinine
NT-pro-BNP	N-terminal B-type natriuretic peptide precursor
COPD	Chronic obstructive pulmonary disease
CHD	Coronary heart disease
Ca	Calcium
Hb	Hemoglobin
PH	Pulmonary hypertension
HFmrEF	Heart failure with mildly reduced ejection fraction
HFrEF	Heart failure with reduced ejection fraction.
